# PBPK modeling to evaluate maximum tolerated doses: A case study with 3-chloroallyl alcohol

**DOI:** 10.3389/fphar.2023.1088011

**Published:** 2023-02-22

**Authors:** Rory P. Conolly, Harvey J. Clewell, Martha M. Moore, Jerry L. Campbell, Wanyun Cheng, R. Robinan Gentry

**Affiliations:** ^1^ Ramboll US Corporation, Monroe, LA, United States; ^2^ Martha M. Moore LLC, Little Rock, AR, United States; ^3^ UPL Limited, Inc, Durham, NC, United States

**Keywords:** PBPK, MTD, genotoxicity, reduction, 3-chloroallyl alcohol

## Abstract

**Introduction:** A physiologically based pharmacokinetic (PBPK) model for 3-chloroallyl alcohol (3-CAA) was developed and used to evaluate the design of assays for the *in vivo* genotoxicity of 3-CAA.

**Methods:** Model development was supported by read across from a published PBPK model for ethanol. Read across was motivated by the expectation that 3-CAA, which like ethanol is a primary alcohol, is metabolized largely by hepatic alcohol dehydrogenases. The PBPK model was used to evaluate how two metrics of tissue dosimetry, maximum blood concentration (Cmax; mg/L) and area under the curve (AUC; mg-hr/L) vary with dose of 3-CAA and with dose route (oral gavage, drinking water).

**Results:** The model predicted that oral gavage results in a 6-fold higher Cmax than the same dose administered in drinking water, but in similar AUCs. Predicted Cmax provided the best correlation with severe toxicity (e.g., lethality) from 3-CAA, consistent with the production of a reactive metabolite. Therefore, drinking water administration can achieve higher sustained concentration without severe toxicity *in vivo*.

**Discussion:** This evaluation is significant because cytotoxicity is a potential confounder of mutagenicity testing. The PBPK model can be used to ensure that studies meet OECD and USEPA test guidelines and that the highest dose used is not associated with severe toxicity. In addition, PBPK modeling provides assurance of target tissue (e.g., bone marrow) exposure even in the absence of laboratory data, by defining the relationship between applied dose and target tissue dose based on accepted principles of pharmacokinetics, relevant physiology and biochemistry of the dosed animals, and chemical-specific information.

## Highlights

• A PBPK model for 3-chloroallyl alcohol (3-CAA) was developed to evaluate the need for an additional *in vivo* genotoxicity assay of 3-CAA conducted by oral gavage instead of in drinking water.
• The model predicted that, at the same administered dose, oral gavage produces a 6-fold higher Cmax than drinking water, but a similar AUC, and that Cmax was more strongly correlated with severe toxicity and lethality.
• Modeling of potential assay designs indicated that an oral gavage study could not increase internal exposure to 3-CAA above that achieved in the drinking water study without leading to overt toxicity.
• The PBPK modeling also demonstrated that, despite the absence of direct tissue data, bone marrow exposure was achieved in the drinking water study.

## 1 Introduction

3-Chloroallyl alcohol (3-CAA) is a metabolite of the herbicide clethodim and of the soil fumigant 1,3-dichloropropene (1,3-DCP). 3-CAA glucoside occurs in leafy crops as a metabolite of clethodim. 3-CAA is the aglycon of 3-chloroallyl alcohol glucoside (EFSA, 2019). 3-CAA is also found in 1,3-DCP contaminated groundwater (EFSA, 2018). In studies of its environmental fate, 3-CAA exhibits low persistence but high mobility in soil (EFSA, 2018). 3-CAA was not detected in any of five regions in the USA above its level of quantification (0.05 μg/L) (van Wesenbeeck and Knowles, 2019) and was found only in trace amounts in an EU monitoring program with reported concentration lower than the level of quantification (0.05 μg/L) (van Wesenbeeck and Knowles, 2019).

As 3-CAA is a metabolite of multiple pesticides and can be found in the environment, a wide range of tests have been conducted to comply with global regulations and to evaluate its potential genotoxicity. 3-CAA was clearly negative in the Ames test (EFSA, 2018), indicating that 3-CAA is unlikely to induce point mutations *in vivo*. 3-CAA was also evaluated for its ability to induce mutations in a mouse lymphoma assay (MLA); the 3-CAA-induced mutant frequency was very small, and both small and large colony mutants were induced in approximately equal frequencies (EFSA, 2018), suggesting that 3-CAA induces, at most, only a very weak mutagenic response. Because the MLA assay can detect both point mutations and chromosomal mutations, appropriate *in vivo* follow up assays include the bone marrow micronucleus (MN) and the transgenic gene mutation assay. In an *in vivo* study of 3-CAA genotoxic potential, its ability to induce micronuclei (MN) in mouse bone marrow was evaluated in male and female mice by single oral gavage on two consecutive days using 0, 31.25, 62.5, and 125 mg/kg body weight. While there was no statistically significant increase in the number of MN in the treated mice, there was also no decrease in the number of polychromatic erythrocytes (PCEs) for the 3-CAA treated mice that would provide evidence of target tissue exposure (EFSA, 2018).

One of the goals of this analysis was to demonstrate that alternative evidence for target tissue exposure for the MN assay can be provided by PBPK modeling. Another goal of this effort was to evaluate the evidence for whether a MTD was achieved in a transgenic genotoxicity rat (TGR) study (Young, 2020). This TGR study was designed to investigate if 3-CAA induces mutations and/or MN in male transgenic Fischer Big Blue^®^ 344 rats. The test substance was administered *via* drinking water at targeted dose levels of 0, 10, 30, and 100 mg/kg/day for 29 days. The actual dose levels achieved, based on water consumption, were calculated to be 0, 9.5, 28.9, and 83.9 mg/kg/day. The dose selection for the 3-CAA TGR study was based on the results of the Crissman et al. (1999) study, which was conducted by the same route (drinking water), for a similar duration (28 days for Crissman et al., 1999 vs. 29 days for the TGR study), and at similar achieved doses (61.9–71.6 mg ingested/kg/day at the highest dose in the 28-day study vs. 83.9 mg ingested/kg/day in the TGR study), and essentially the same strain of rats (Big Blue Fischer 344 rats *versus* standard Fischer 344 rats). The 28-day study identified liver as the target tissue for 3-CAA with observations of hepatocellular hypertrophy and individual cell necrosis at the highest dose, therefore, the TGR study adopted the same nominal doses of 0, 10, 30, and 100 mg/kg/day. No evidence of effects of 3-CAA on mutant frequency and micronucleus induction were observed in the TGR study. Clinical signs were reported in the TGR study and included significantly decreased water and feed consumption, significantly decreased final body weights, and increased relative liver weight. However, no histopathology was performed in this study, so it was not possible to provide direct evidence that the increased relative liver weight was the result of hepatotoxicity from 3-CAA.

Many guidelines for the design of *in vivo* assays require that a maximum tolerated dose (MTD) is achieved. The MTD is intended to ensure that the testing includes a dose high enough to cause at least mild systemic toxicity (USEPA, 2005; OECD, 2016; OECD, 2020). In studies with laboratory animals, the MTD can be defined as not affecting survival, causing changes in body weight gain, and, at most, minimal signs of overt toxicity (OECD, 2002). For the genotoxicity assays, the OECD Test Guidelines were written with the goal of attaining sufficient toxicity to assure that observed negative responses are reflective of true lack of genotoxic activity. It is also important to note, however, that in some cases, the toxicity occurring at the MTD may differ from the target tissue toxicities that the test is designed to detect. For example, the high dose in an *in vivo* transgenic gene mutagenicity study might produce hepatotoxicity that is independent of any mutagenic effect. A second, related concern is the possibility that the target outcome for a study, such as an observed increase in the number of mutations, could be a mechanistic consequence of the systemic toxicity, hepatotoxicity. If this were the case, a mutagenic outcome observed only at the MTD may not be a direct mutagenic effect of the substance being tested, but rather a consequence of concurrent cytotoxicity. Since various toxicity testing guidelines do not always identify quantitative metrics of toxicity that can be used to specify exactly when a high dose is a MTD, expert judgment should be part of the evaluation.

The present work describes the development of a physiologically based pharmacokinetic (PBPK) model for 3-CAA in the rat and use of this model to evaluate pharmacokinetic aspects of the designs of several tests of the *in vivo* toxicity of 3-CAA. The main goals of this work were to demonstrate 1) development of a PBPK model using read-across information from a structurally related chemical and 2) how PBPK modeling can help to characterize the relationships between applied dose, target tissue dose, and MTD for studies intended to support regulatory evaluation of 3-CAA. An additional goal was to describe how these pharmacokinetic analyses can inform decision making about the design of studies, such as those designed for mutagenicity testing for 3-CAA, helping to ensure that the probability of detecting potential mutagenicity is maximized while the likelihood that excessive concurrent toxicity, e.g., cytotoxicity leading to deregulation of cell homeostasis (Gentile et al., 2017), that might compromise the interpretation of the data is minimized. These results are relevant to guideline toxicity testing of 3-CAA and to other chemicals where the test data must be evaluated at both the MTD and at lower doses where concurrent cytotoxicity may or may not be a concern. In general, PBPK modeling represents a cost- and time-effective approach to assist agencies by improving chemical management by designing the most effective animals studies and potentially reducing animal use.

## 2 Methods

**FIGURE 1:**
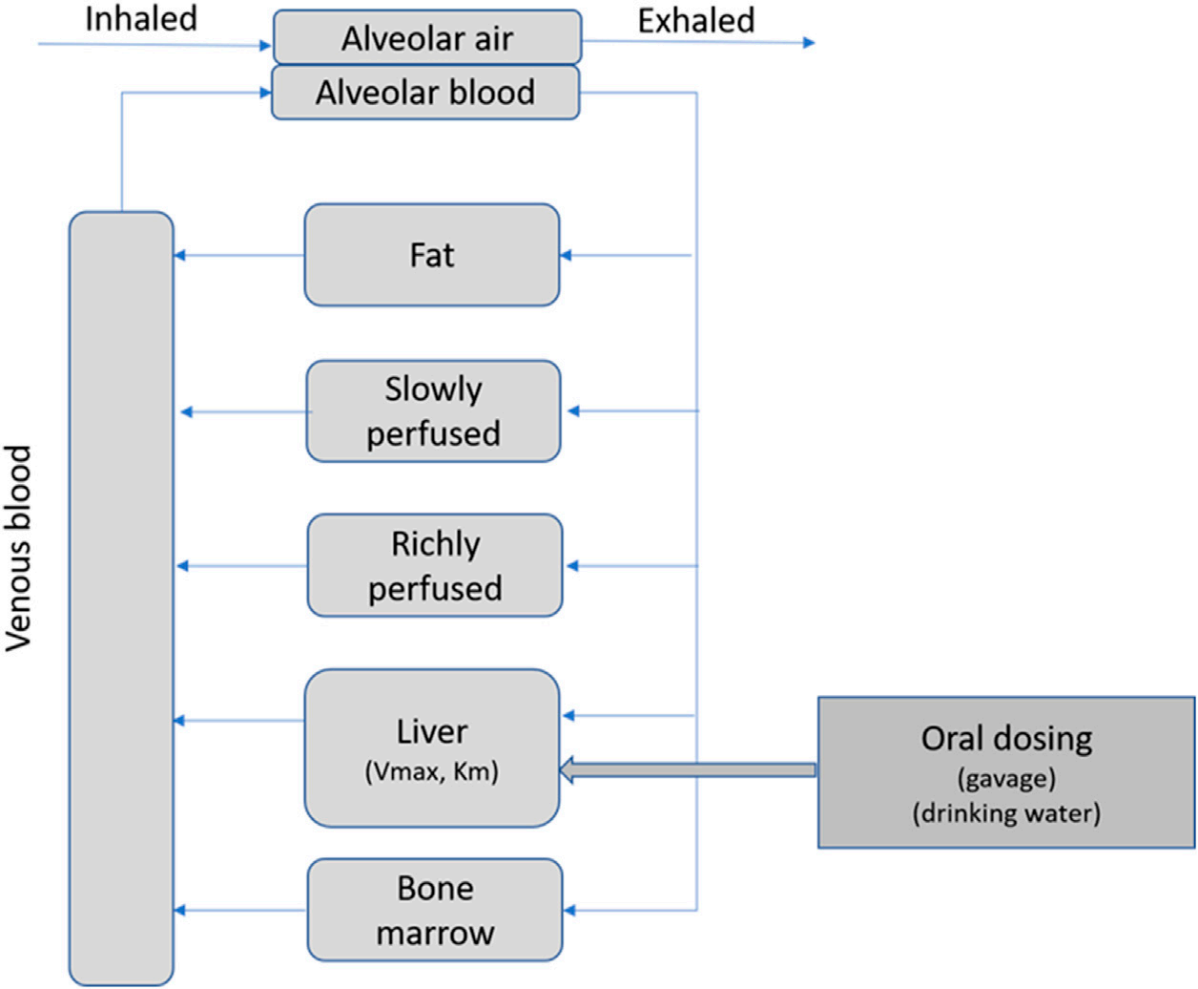
PBPK model for 3-CAA.

The PBPK model was designed to accommodate oral dosing of 3-CAA by drinking water or oral gavage, absorption of 3-CAA from the oral dosing compartment to the liver, saturable metabolism of 3-CAA in the liver, exhalation of 3-CAA *via* a blood-air interface, and distribution of 3-CAA by blood flow to bone marrow, liver, fat, richly perfused, and slowly perfused compartments (Figure 1). Standard physiological parameters for blood flow and tissue volumes for adult rats were used (Perleberg et al., 2004).

3-CAA is structurally similar to allyl alcohol, which only differs from 3-CAA by the absence of a chlorine in the 3-position. The primary toxicity of allyl alcohol is periportal liver toxicity that results from its metabolism by alcohol hydrogenase (ADH), and at lower concentrations cytochrome.

(CYP) oxidation, to acrolein, a highly reactive, cytotoxic compound (Badr, 1991). The equivalent metabolite from 3-CAA is 3-chloroacrolein, which, based on the similarity of structure, is also reactive and cytotoxic (Eder and Dornbusch, 1988).

While physiological parameters for use in a rat PBPK model are readily available, estimates of the Michaelis-Menten parameters Vmax and Km for metabolism of 3-CAA by ADH in rats are not. However, Pastino and Conolly (2000) described a PBPK model for ethanol in rats. This model provides Vmax and Km values for the hepatic metabolism of ethanol by ADH. These values, adjusted for the difference in molecular weight between ethanol and 3-CAA, were used as initial values of Vmax and Km values for the PBPK modeling of 3-CAA, as described further in Results.

The blood-air and tissue:blood partition coefficients (Table 1) were estimated using the method included in the IndusChemFate PBTK model (ver. 2.0) as reported by Jongeneelen and Ten Berge (2011). This approach uses molecular weight, vapor pressure and water solubility as inputs. Tissue:blood partition coefficients were estimated using the algorithms reported by DeJongh et al. (1997), which are tissue specific, using the octanol:water partition coefficient at pH seven reported in Bendig and Paschke (2020).

**TABLE 1:** 3-CAA partition coefficients

Blood-air	1492
Liver:blood	0.95
Fat:blood	1.47
Richly perfused:blood	0.95
Slowly perfused:blood	0.64
Bone marrow:blood	1.06

**FIGURE 2:**
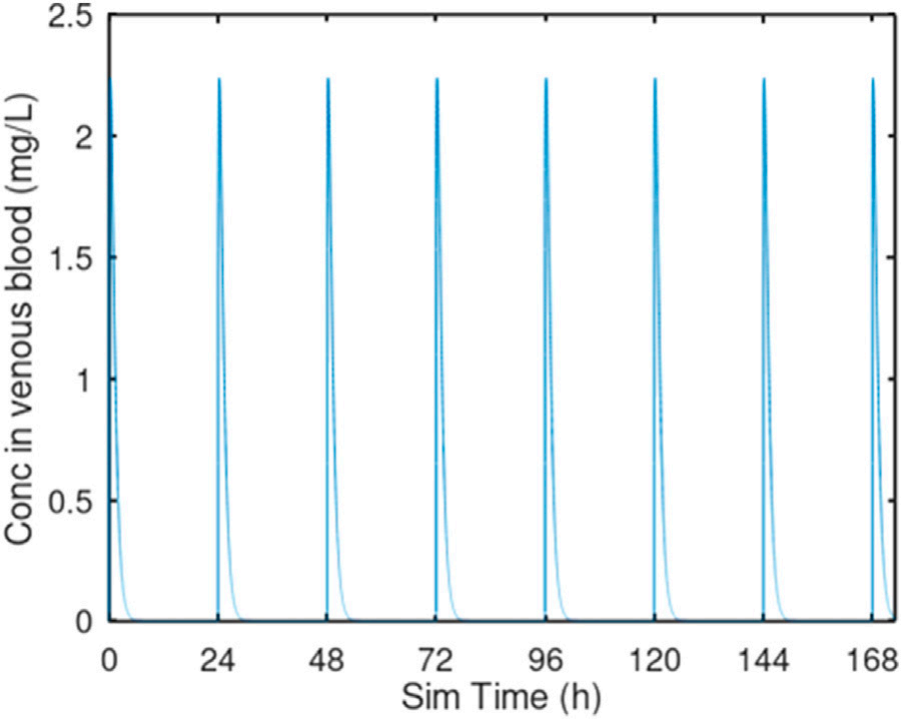
Oral gavage dosing. Predicted venous blood concentration for 7 days of oral gavage dosing at 10 mg/kg/day. Each oral gavage dose was simulated as a 10 s infusion into the oral dosing compartment.

**FIGURE 3:**
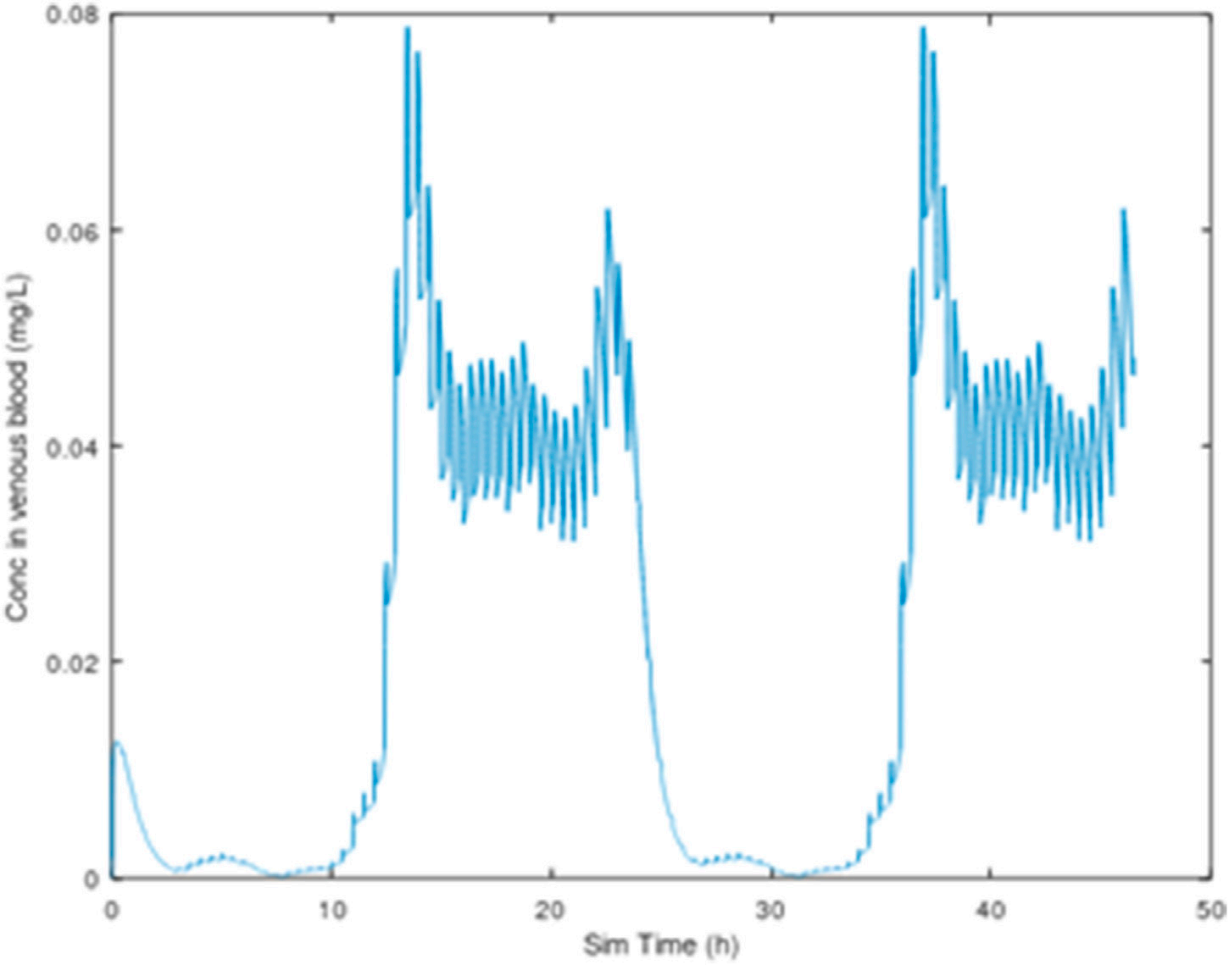
3-CAA in venous blood, 25 mg/kg/day, with dosing (sipping) every 30 min, 2-day simulation, using the drinking water consumption behavior described by Yuan (1993).

Oral gavage dosing was described as a single daily infusion into the dosing compartment, with the infusion lasting 15 s (Figure 2). Drinking water dosing was guided by data from Yuan (1993), who measured drinking water consumption in male Sprague-Dawley rats at half-hour intervals for 24 h. Simulated drinking water dosing involved 48 infusions into the dosing compartment, spaced 0.5 h apart, to cover the 24-h day. Each drinking water infusion lasted 15 s (Figure 3). For both oral gavage and drinking water, the rate of absorption of 3-CAA from the dosing compartment was determined by a first-order rate constant with a value of 1.0/hr.

PBPK model code was written in the public domain scientific programming language GNU Octave 6.2.0 (Eaton et al., 2021). The model code consists of a set of text files that are included as (Supplementary Material–PBPK model.zip).

## 3 Results

### 3.1 PBPK modeling of 3-CAA

As noted previously, 3-CAA is a structural analog of allyl alcohol, which is metabolized by alcohol dehydrogenase (ADH) (Ohno et al., 1985). Hansen and Bartels (2000) examined both 3-CAA and 3-chloroacrylic acid; 3-chloroacrylic acid would be formed from 3-CAA *via* metabolism by ADH. The Hansen and Bartels (2000) study documents extensive metabolism of 3-CAA, which is consistent with systemic availability, as described by the PBPK model and with the expected metabolism by ADH. Pastino and Conolly (2000) used PBPK modeling to study ethanol metabolism in rats. They used Michaelis-Menten parameters, Vmax and Km, for the saturable hepatic metabolism of ethanol by ADH, of 110.64 mg/h and 23 mg/L (Km). Adjusting for the difference in molecular weight between ethanol and 3CAA, (46.07 g/mol vs. 92.52 g/mol, respectively), the equivalent Michaelis-Menten values for 3-CAA are Vmax = 222.16 mg/h and Km = 46.1 mg/L. For the present analysis, Vmax, which is associated with a specific body weight, was converted to the body weight scalable quantity VmaxC using the conventional approach for within-species scaling, which reflects the relationship between liver volume and enzyme capacity:

$$VmaxC=Vmax∙{BW}^{0.75}$$

Using body weight = 0.225 kg, VmaxC for 3-CAA is 680 mg/h. Km is not a function of body weight and remains at 46.1 mg/L. Since measured values for the 3-CAA Vmax and Km were not available, the values based on read-across from ethanol were used a starting points for scans of model behavior across a range of VmaxC and Km values (Table 2). While the factor of 3 used to construct Table 2 is arbitrary, it provided a range of values that were used to determining if PBPK model predictions of venous blood Cmax and AUC values were sensitive to variation in these parameter values and to a range of doses of 3-CAA as specified below.

**TABLE 2:** Metabolic parameters for 3-CAA.

VmaxC (mg/hr/kg^0.75^)				
226	452	904	1808	Pastino & Conolly: 680
Km (mg/L)				
15.3	30.6	61.2	122	Pastino & Conolly: 46.1

Note that VmaxC = 226 and Km = 15.3 are each 1/3 of the values identified by read across from ethanol (680 and 46.1, respectively; Pastino and Conolly, 2000). These minimum values are then progressively increased by a factor of 2 to obtain the remaining values.

Venous blood Cmax and AUC values for drinking water and oral gavage dosing for all possible combinations of the VmaxC and Km values (Table 2) were obtained for simulated oral gavage and drinking water doses of 10, 30, 60, and 83.9 mg/kg/day (to span the range of doses in the TGR study) for 7 days (to achieve steady-state conditions) (Supplementary Appendix SA1). All values of the ratio Cmax_oral gavage/Cmax_drinking water fell in the range 4.80–6.91 while the values for AUC_oral gavage/AUC drinking water fell in the range 0.99–1.15. Thus, for the values of VmaxC and Km that were evaluated, oral gavage dosing was consistently predicted by the PBPK model to result in a 5–6-fold higher Cmax than the same dose given in drinking water. AUC values for oral gavage and drinking water dosing were similar. The predicted systemic bioavailability for the simulations in Supplementary Appendix SA1 ranges from as low as 2.1% (for the highest value of VmaxC and lowest value of Km) to as high as 60% (for the lowest value of VmaxC and the highest value of Km).

The results of the Cmax-AUC comparison described above can be used to identify plausible values of VmaxC and Km that maximize the metabolism of 3-CAA. These are:

$$VmaxC=1808\;mg/hr/{kg}^{0.75}$$

Km=15.0 mg/L

Note that these values are the maximum VmaxC and the minimum Km that were evaluated in the comparisons (Table 2) and maximize the metabolism of 3-CAA and thereby the production of reactive metabolites. One area of note is in the estimation of systemic bioavailability. The predicted bioavailability for all of the simulations in Table 3 is 2.1%, indicating that most of the absorbed dose is metabolized by first pass metabolism. The small percentage of the absorbed dose that is predicted to escape first pass metabolism would be distributed throughout the body to all perfused tissues (Figure 1).

**TABLE 3:** Predictive Simulations for 28 days of dosing of 3-CAA.

Route of administration						
	Dose (mg/kg/day)	Cmax (mg/L)	AUC (mg-hr/L)	Bioavailability(%)		Reference
Oral gavage	25	0.096	3.89		2.1	Carney and Liberacki(1999)
Oral gavage*	75	0.294	11.8		2.1	Liberacki (1999)
Drinking water	61.9	0.041	9.71		2.1	Crissman et al. (1999)
Drinking water	75	0.049	11.7		2.1	---
Drinking water	83.9	0.055	13.1		2.1	Young 2020

*Predictive Simulations:* Several predictive simulations are presented (Table 3) for dose administration over a period of 28 days, using the PBPK model configured with VmaxC = 1808 mg/h and Km = 15.0 mg/L.

**FIGURE 4:**
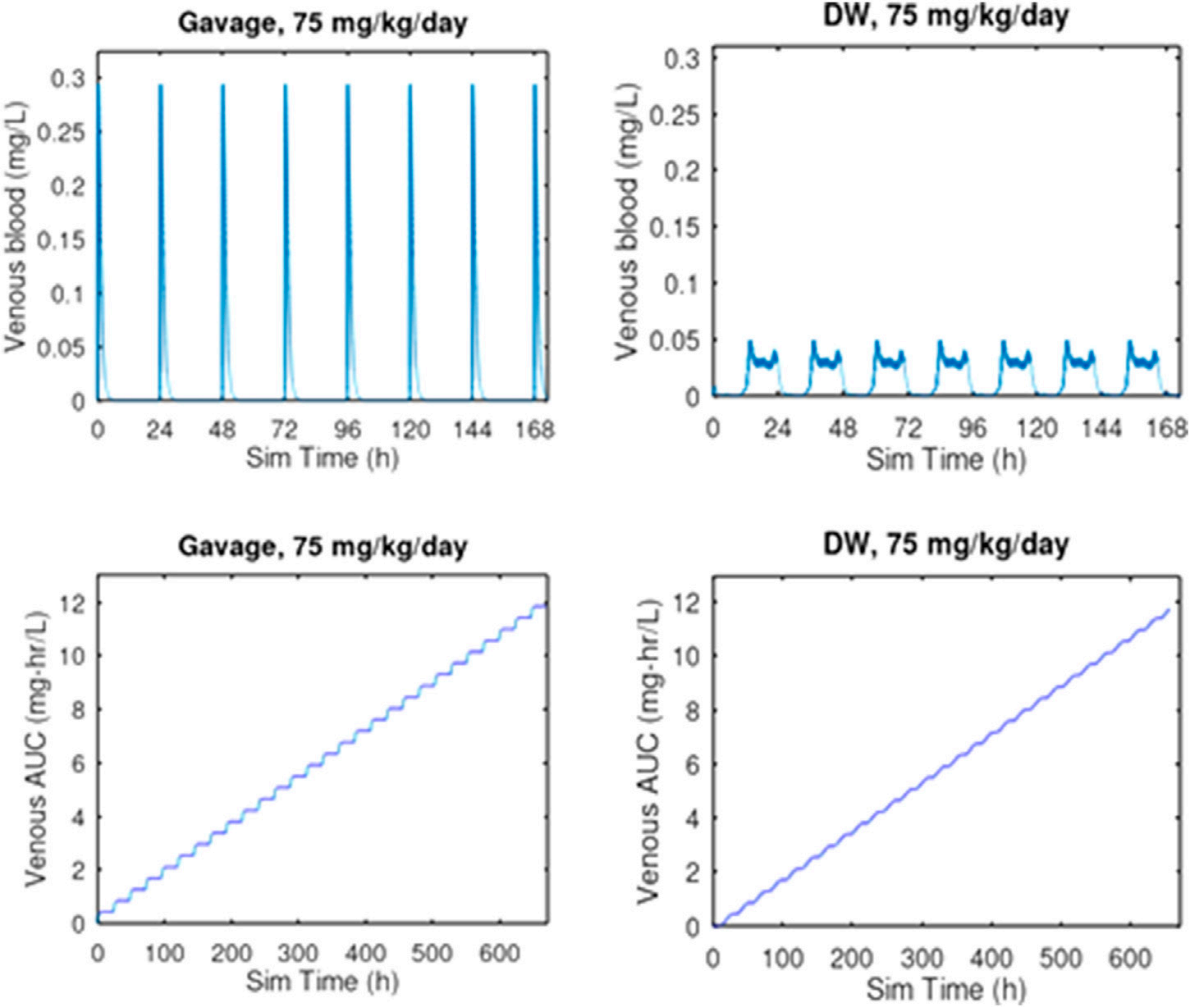
PBPK comparison of Cmax and AUC for oral gavage and drinking water exposure at 75 mg/kg/day for 28 days. Oral gavage dosing is predicted to result in a 5.9-fold greater venous blood concentration than the same daily dose given in drinking water. Given that this dose of 75 mg/kg/day was lethal to 2 of 10 rats by oral gavage after 2 days of exposure (Liberacki, 1999) while all 6 rats survived 29 days of exposure to 83.9 mg/kg/day by drinking water administration (Young, 2020), these results suggest that the considerably higher Cmax associated with oral gavage dosing is a key determinant of the toxicity of 3-CAA.

• The highest dose tested in a 15-day developmental study conducted with 3-CAA administered by aqueous gavage in CD rats was limited to 25 mg/kg/day (Carney and Liberacki, 1999), because administration of higher doses (75 mg/kg/day) proved to be lethal in a preliminary range-finding study (death of 2/10 animals after receiving two doses) (Liberacki, 1999). Therefore, this dose, administered daily by oral gavage, was compared with the same dose administered daily by drinking water. These predictive simulations indicate that, at 75 mg/kg/day, oral gavage dosing results in 5.9-fold higher Cmax than drinking water, but in similar AUC. (see also Figure 4).
• the study by Liberacki (1999), the PBPK predicted ratio (average 3-CAA concentration in bone marrow)/Cmax was 0.336 for drinking water dosing and 0.057 for oral gavage dosing. Thus, not only does gavage dosing result in a higher Cmax than drinking water dosing, the profile over time of the tissue concentration is highly skewed with oral gavage dosing, while the profile for drinking water dosing in much less extreme.
• Young (2020) and Crissman et al. (1999) administered 3-CAA by drinking water, whereas Carney and Liberacki (1999) administered 3-CAA by oral gavage. The PBPK model was used to predict the Cmax and AUC associated with the highest doses administered in each of these studies (Table 3).
• Cmax following oral gavage administration of 25 mg/kg/day was predicted to exceed Cmax following drinking water exposure to 83.9 mg/kg/day. AUC for drinking water following exposure to 83.9 mg/kg/day was predicted to be about three times greater than AUC following oral gavage administration of 25 mg/kg/day (Table 3).

3-CAA dosed by either oral gavage or in drinking water is expected to be well absorbed from the GI tract into the hepatic portal circulation and then diffuse from the portal blood into the hepatic parenchyma. Due to its structural analogy with allyl alcohol, 3-CAA is expected to be metabolized in the liver, primarily by ADH, to a reactive metabolite, 3-chloroacrolein. 3-CAA that escapes hepatic first-pass metabolism would leave the liver in venous blood and be distributed *via* arterial blood flow to the rest of the body (Figure.1).

PBPK modeling predicts that much of the absorbed dose of 3-CAA is metabolized in the liver. The hepatic metabolism of 3-CAA to 3-chloroacrolein offers a probable mechanistic explanation for the observed hepatotoxicity. The intensity of the toxicity is expected to be proportional to the rate of metabolism (Andersen et al., 1987; Liao et al., 2007).

### 3.2 *In Vivo*, transgenic rodent mutagenicity testing of 3-CAA

A TGR study was conducted that investigated the potential effects of 3-CAA on mutant frequency and micronucleus induction in male transgenic Fischer Big Blue^®^ 344 rats (Young, 2020). The test substance was administered *via* drinking water at targeted dose levels of 0, 10, 30, and 100 mg/kg/day for 29 days. The actual dose levels achieved, based on water consumption, were calculated to be 0, 9.5, 28.9, and 83.9 mg/kg/day. The dose selection for the transgenic (TGR) study was based on an earlier 28-day toxicity study (Crissman et al., 1999) using the same targeted dose levels, same route of administration, similar length of study and essentially the same strain of rats (Big Blue Fischer rats *versus* standard Fischer F344 rats) (See Table 4). The highest dose tested in the TGR study (nominal dose of 100 mg/kg/day, 83.9 mg consumed/kg/day, Young, 2020) was associated with evidence of significant toxicity. Relative liver weight was increased, while other relative organ weights were unaffected, suggesting the possibility of hepatomegaly.

**TABLE 4:** 3-CAA partition coefficients

	1999 Repeat Toxicity	2020 TGR Study
Author	Crissman et al., 1999	Young 2020
Dosing route	Drinking water	Drinking water
Animal	F344 rat, male & female	Transgenic F344 rat, male
Dosing days	28	29
Nominal dose (mg/kg/day)	0, 10, 30, 100	0, 10, 30, 100
Achieved dose (mg/kg/day)	Male: 0, 10.1, 27.0, 61.9	0,9.5, 28.9, 83.9
	Female: 0, 10.4, 27.0, 71.6	
Systemic toxicity	Decreased body weight gain, increased relative liver weight, liver toxicity including necrosis at high dose	Decreased body weight gain, increased relative liver weight, at high dose

None of the tested doses (nominally 10, 30, 100 mg/kg/day) in the Young (2020) study were associated with change in numbers of micronucleated reticulocytes or in mutant frequency in the *cII* gene in either liver or bone marrow. The negative result for genotoxicity in liver is particularly notable since the PBPK modeling predicts high first pass clearance of 3-CAA. Assuming metabolism by ADH, this high first pass clearance would be associated with production of 3-chloroacrolein, the presumed reactive metabolite of 3-CAA that would be responsible for any potential mutagenic effect of 3-CAA.

## 4 Discussion

Liver was identified as a target organ for 3-CAA and sufficient exposure was indicated by the observations of increased relative liver weight and hepatocellular hypertrophy and necrosis (Crissman et al., 1999; Young, 2020). The OECD guideline for the micronucleus assay (OECD, 2016) requires demonstration of adequate exposure of the target tissue (e.g., bone marrow toxicity measured with decreased PCEs). The absence of 3-CAA bone marrow toxicity could be interpreted as indicating that there was no 3-CAA exposure to the bone marrow. However, both the available pharmacokinetic data (Hansen and Bartels, 2000) and the PBPK analysis considered in detail below provide assurance of bone marrow exposure.

Hansen and Bartels (2000) found that ^14^C-3-CAA dosed orally in rats (5 or 75 mg/kg) was extensively metabolized, with 85%–94% of the administered radioactivity recovered. 50%–52% of the dose was recovered as expired ^14^C-CO_2_. These data demonstrate that 3-CAA is well absorbed from the GI tract.

The PBPK model defines the relationship between applied dose and target tissue dose in terms of accepted principles of pharmacokinetics (absorption, distribution, metabolism, excretion–ADME), relevant physiology and biochemistry of the dosed animals, and chemical-specific information such as partition coefficients and metabolism rate parameters. The PBPK model for 3-CAA, and other published PBPK models that include explicit bone marrow compartments (e.g., Perleberg et al., 2004; Knutsen et al., 2013) are based on these principles and all show that a chemical that can be absorbed from its site of first contact with the body and then reach circulating blood, will be delivered to all tissues that are perfused by arterial blood.

Genotoxicity studies such as the micronucleus assay require demonstration of target tissue exposure using biomarkers such as a depression of the immature to mature erythrocyte ratio or measurement of the plasma or blood levels of the test substance (OECD, 2016). The PBPK model for 3-CAA predicts that, even though most of the dose absorbed from the dosing compartment (i.e., the GI tract) is metabolically cleared by hepatic first pass metabolism, some unaltered 3-CAA, at least 2.1% of the absorbed dose, escapes first pass metabolism and leaves the liver in the venous blood. 3-CAA is then distributed to all tissues that are perfused by the arterial blood, including the bone marrow. Thus, although a decrease in the immature to mature erythrocyte ratio was not observed after 3-CAA exposure (Young, 2020), PBPK simulation indicates that 3-CAA is bioavailable to bone marrow after oral dosing.

PBPK modeling analysis of the study by Liberacki (1999) indicates that lethality seen in rats dosed with 75 mg/kg/day 3-CAA by oral gavage is associated with the Cmax of 0.294 mg/L. The study by Young (2020) dosed rats by drinking water with 83.9 mg/kg/day for 28 days and saw no lethality. Predicted Cmax for Young (2020) was 0.041 mg/L, about 13% of the predicted Cmax for Liberacki (1999).

The PBPK model used in this analysis describes the total rate of metabolism of 3—CAA but the model does not track the concentration of the metabolite(s) over time because there are no data that could support parameter estimation. For the present, we are constrained to limit our considerations to the kinetics of 3-CAA and its rate of metabolism. Rate of metabolism has been used previously for chemicals with a mode of action based on toxicity of reactive metabolite(s) (Andersen et al., 1987, Clewell et al., 2019). As noted in the text, it is expected that 3-CAA is metabolized to 3-chloroacrylic acid, which would be highly reactive and unlikely to diffuse out of the tissue where it is generated. Moreover, in our PBPK modeling, the initial VmaxC and Km we used were identified by read-across from the PBPK model for ethanol, and we do not have any such read-across information that could guide the parameterization of rate of metabolism in extrahepatic tissues such as bone marrow.

Tt is important to note that our evaluation of how changes in the values of VmaxC and Km affect predicted Cmax and AUC for 3-CAA (Supplementary Appendix SA1) found that metabolism of 3-CAA was linear with these changes over a relevant range of does. In other words, metabolism was not saturating, which would have introduced a non-linearity into the relationship dose to amount metabolized. Since the production of the reactive metabolite is a linear function of the concentration of 3-CAA, it’s reasonable to use the 3-CAA Cmax and AUC as metrics of the rate of production of reactive metabolites. This differs from the case for chloroform, for example, which is metabolized primarily by CYP2E1. In that case, the rate of metabolism has a non-linear relationship with the concentration of chloroform, because the metabolism of chloroform by CYP2E1 saturates at relatively low concentrations. However, a toxic dose of chloroform by oral gavage will still tend to be more toxic than the same dose administered by drinking water. Similarly, since the scenarios we are comparing are in the same species and strain, with the same metabolic parameters, we can infer the relationship between the rates of reactive metabolite generation in the liver under different dosing scenarios from the relationship of 3-CAA concentrations.

In contrast to comparing dosing scenarios in the same animal species, when conducting human risk assessments based on animal data, it is critical to use a dose metric that is appropriate for the metabolite(s) of concern to account for differences in the relationship between parent chemical concentration and metabolite production across species, If one is extrapolating a dose-response relationship from rats to humans, for example, and specific responses have been observed at given administered doses, then the highest estimated human risk will be obtained from the *lowest* estimated rat (internal) doses, because then (roughly) response/dose is maximized. Therefore, any species comparison for 3-CAA toxicity would need to use the rate of metabolism dose metric rather than 3-CAA concentration.

**FIGURE 5:**
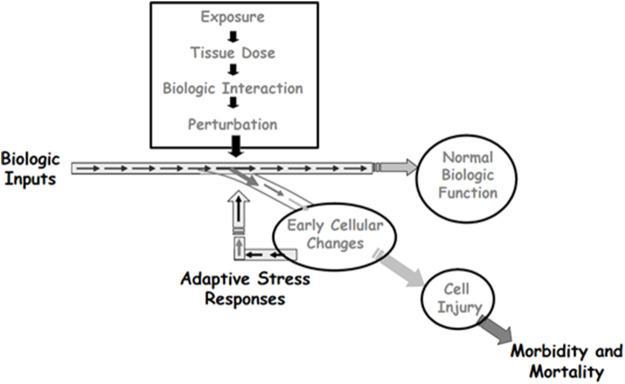
Depiction of how adaptive stress responses can lead to survival of cells that transiently experience toxic stress. When the stress includes reactive species that can damage DNA, cells that survive may carry mutations that are secondary effects of the transient toxicity. Source: Adapted from Andersen, M.E., Dennison, J.E., Thomas, R.S., and Conolly, R.B. New directions in incidence-dose modeling. Trends in Biotechnology 23 (3):122-127. Reprinted with permission; copyright 2005, *Trends in Biotechnology.*

The cytotoxic environment is rich with reactive species (Nakamura et al., 2014; Gentile et al., 2017) and is associated with activation of multiple stress response pathways, including those for DNA damage, inflammation, and oxidative stress (National Research Council, 2007; Simmons et al., 2009). Activation of these pathways allows cells to survive moderate levels of stress and eventually return to normalcy (Figure 5). However, the inflammatory signaling associated with this stress response can itself provide the prerequisite environment for the development of malignancy (O’Byrne and Dalgleish, 2001; Nakamura and Nakamura, 2020). The concern with evaluation of data obtained at the MTD as part of a mutagenicity study is not with the occurrence of apoptosis or necrosis, where the cell dies. Rather, the concern is with recurrent development of a cytotoxic environment that disrupts cellular function without inducing apoptosis or necrosis. (Figure 5). In this case, the reactive species that existed transiently at elevated levels may have damaged DNA or altered the function of the biochemical machinery that replicates DNA and controls cell division. Cytotoxicity can impair the ability of the cell to repair DNA damage and, when coupled with a compensatory increase in cell proliferation, can enhance the fixation of unrepaired DNA adducts, leading to disproportional increases in mutation (Takahashi et al., 2000; Swenberg et al., 2008; Lu et al., 2012; Clewell et al., 2018; 2019). This sustained proliferative environment increases the probability that the steps toward neoplasia will occur (Wolf et al., 2019). In this way, mutations can occur that are an indirect consequence of cytotoxicity, rather than a direct reaction of the compound with DNA or with a regulator of the cell cycle.

The hepatocarcinogenicity of chloroform in rats provides a good example of how differences in Cmax between drinking water and oral gavage exposures can explain differences in genotoxic outcomes. Chloroform dosing by corn oil gavage is hepatocarcinogenic while dosing in drinking water is not (Wolf and Butterworth, 1997). Chloroform is not directly genotoxic but is a potent hepatotoxicant (Reitz et al. (1982). PBPK modeling for chloroform (Corley et al., 1990) shows that the Cmax achieved with corn oil gavage is much higher than that achieved with drinking water administration for doses with similar blood AUC. Thus, cytotoxicity correlates with Cmax, not with AUC, and the hepatocarcinogenicity of chloroform is considered to be secondary to its hepatotoxicity (USEPA, 2001; Boobis, 2010).

The evidence of cytotoxicity in the liver in the previous 3-CAA repeated dose toxicity study (Crissman et al., 1999) identifies the highest dose in the transgenic study (Young, 2020) as an MTD. Use of a higher dose, with the associated probability of an increase in liver toxicity and an increased potential for genotoxicity associated with this liver toxicity, might compromise the usefulness of the genotoxicity assessment. The PBPK modeling analysis of 3-CAA pharmacokinetics by oral gavage and drinking water dosing indicates that conducting a transgenic study using oral gavage of 3-CAA would not increase internal exposure, compared to drinking water administration–the AUCs for the two routes of exposure would be similar. However, the Cmax for oral gavage would be about 6-fold higher than that for drinking water. Moreover, as noted preciously in the analysis of Liberacki (1999), the PBPK predicted ratio (average [3-CAA] in bone marrow)/Cmax was 0.336 for drinking water dosing and 0.057 for oral gavage dosing. Thus, drinking water exposure is predicted to provides tissue exposure for the duration of the study that is closer to the Cmax than does oral gavage dosing. The higher Cmax for oral gavage dosing and the smaller variation over time in blood and tissue concentrations with drinking water exposure suggest that an MTD study using drinking water exposure will achieve more tissue exposure than a gavage MTD study with a similar level of toxicity at the MTD. Furthermore, for 3 CAA, drinking water is the most relevant route of exposure, and the OECD guidelines for both transgenic gene mutation and MN testing recommend using the most relevant route (OECD, 2016; OECD, 2020).

In summary, the TGR study and the larger 3-CAA database were evaluated with consideration of dosimetry predictions provided by the PBPK model for 3-CAA and in light of regulatory agency guidance on the interpretation of MTD studies. PBPK modeling analysis of the design of the TGR study indicates that drinking water administration maximizes tissue exposure, as defined by the AUC, while providing a Cmax associated with mild hepatotoxicity. An alternative study design using oral gavage dosing that would achieve the same AUC was predicted by the PBPK model to be associated with an approximately 6-fold higher Cmax and, therefore, a greater probability of significant hepatotoxicity. This gavage-associated toxicity could limit the ability of the testing to identify genotoxic effects that are independent of cytotoxicity. These results illustrate the value of examining the test compound pharmacokinetics, using *in silico* modeling, to ensure a study design that maximizes tissue exposure (AUC) while helping, by prediction of Cmax, to ensure that the MTD is associated with an appropriate level of systemic toxicity (as described in the TGs). In the present work, the PBPK model for 3-CAA was developed using read across from published studies of related compounds, illustrating the use of a novel new approach methodology relevant to design of *in vivo* studies of mutagenicity. Regulatory implementation of these new approaches would be accelerated by updating existing regulations to address the capability of PBPK modeling to provide evidence of target tissue exposure.

## Data Availability

The original contributions presented in the study are included in the article/Supplementary Material, further inquiries can be directed to the corresponding author.

## References

[B2] Andersen M. E. Clewell H. J. Gargas M. L. Smith F. A. Reitz R. H. (1987). Physiologically-based pharmacokinetics and the risk assessment process for methylene chloride. Toxicol. Appl. Pharmacol. 87, 185–205. 10.1016/0041-008x(87)90281-x 3824380

[B4] Badr M. Z. (1991). Periportal hepatotoxicity due to allyl alcohol: A myriad of proposed mechanisms. J. Biochem. Toxicol. 6 (1), 1–5. 10.1002/jbt.2570060102 1880785

[B5] Bendig P. Paschke J. (2020). Determination of n-octanol/water partition coefficients (log p_ow_ values) using RP-HPLC/UV (OECD 117) for clethodim and metabolites. Ulm, Germany: EAG Laboratories GmbH. EAG Laboratories ID: P 5124 G.

[B6] Boobis A. R. (2010). Mode of action considerations in the quantitative assessment of tumour responses in the liver. Basic Clin. Pharmacol. Toxicol. 106 (3), 173–179. 10.1111/j.1742-7843.2009.00505.x 20030633

[B7] Carney E. W. Liberacki A. (1999). 3-Chloroallyl alcohol: Oral gavage developmental toxicity study in CD rats. Indianapolis: Dow AgroSciencesIndiana. Study number 981204.

[B8] Clewell H. J. Yager J. W. Greene T. B. Gentry P. R. (2018). Application of the adverse outcome pathway (aop) approach to inform mode of action (moa): A case study with inorganic arsenic. J. Toxicol. Environ. Health A 81 (18), 893–912. 10.1080/15287394.2018.1500326 30230972

[B9] Clewell R. A. Thompson C. M. Clewell H. J. 3rd (2019). Dose-dependence of chemical carcinogenicity: Biological mechanisms for thresholds and implications for risk assessment. Chem. Biol. Interact. 301, 112–127. 10.1016/j.cbi.2019.01.025 30763550

[B44] Clewell H. J. 3rd Campbell J. L. Van Landingham C. Franzen A. Yoon M. Dodd D. E. (2019). Incorporation of in vitro metabolism data and physiologically based pharmacokinetic modeling in a risk assessment for chloroprene. Inhal Toxicol. 13-14, 468–483. 10.1080/08958378.2020.1715513 31992090

[B10] Corley R. A. Mendrala A. L. Smith F. A. Staats D. A. Gargas M. L. Conolly R. B. (1990). Development of a physiologically based pharmacokinetic model for chloroform. Toxicol. Appl. Pharmacol. 103, 512–527. 10.1016/0041-008x(90)90324-n 2339423

[B11] Crissman J. W. Dryzga M. D. Cieszalk F. S. (1999). 3-Chloroallyl alcohol: 4-week repeated dose drinking water toxicity study in fischer 344 rats. Indianapolis, Indiana: Dow AgroSciences (DAS) LLC. Study Number 981194.

[B12] DeJongh J. Verhaar H. J. Hermens J. L. (1997). A quantitative property-property relationship (QPPR) approach to estimate *in vitro* tissue-blood partition coefficients of organic chemicals in rats and humans. Archives Toxicol. 72, 17–25. 10.1007/s002040050463 9458186

[B13] Eaton J. W. Bateman D. Hauberg S. Wehbring R. (2021). GNU Octave version 6.2.0 manual: A high-level interactive language for numerical computations. Available at: https://www.gnu.org/software/octave/doc/v6.2.0/.

[B14] Eder E. Dornbusch K. (1988). Metabolism of 2,3-dichloro-1-propene in the rat. Consideration of bioactivation mechanisms. Drug Metab. Dispos. 16 (1), 60–68. 2894957

[B1] EFSA (European Food Safety Authority), Anastassiadou M. Brancato A. Carrasco Cabrera L. Ferreira L. Greco L. Jarrah S. (2019). Review of the existing maximum residue levels for clethodim according to Article 12 of Regulation (EC) No 396/2005. EFSA J. 17 (5), e05706. 10.2903/j.efsa.2019.5706 32626327 PMC7009186

[B3] EFSA (European Food Safety Authority), Arena M. Auteri D. Barmaz S. Brancato A. Brocca D. Bura L. (2018). Peer review of the pesticide risk assessment of the active substance (EZ)-1,3-dichloropropene. EFSA J. 16 (11), e05464. 10.2903/j.efsa.2018.5464 32625746 PMC7009673

[B15] Gentile F. Arcaro A. Pizzimenti S. Daga M. Cetrangolo G. P. Dianzani C. (2017). DNA damage by lipid peroxidation products: Implications in cancer, inflammation and autoimmunity. AIMS Genet. 4 (2), 103–137. 10.3934/genet.2017.2.103 31435505 PMC6690246

[B16] Hansen S. C. Bartels M. J. (2000). Pharmacokinetics and metabolism of either ^14^C-3-chloroallyl alcohol or ^14^C-3-chloroacrylic acid following a single oral administration to male Fisher 344 rats. Midland MI: Dow Chemical. Study ID 991136.

[B17] Jongeneelen F. J. Ten Berge W. F. (2011). A generic, cross-chemical predictive PBTK model with multiple entry routes running as application in MS Excel; Design of the model and comparison of - predictions with experimental results. Ann. Occup. Hyg. 55, 841–864. 10.1093/annhyg/mer075 21998005

[B18] Knutsen J. S. Kerger B. D. Finley B. Paustenbach D. J. (2013). A calibrated human PBPK model for benzene inhalation with urinary bladder and bone marrow compartments. Risk Anal. 33, 1237–1251. 10.1111/j.1539-6924.2012.01927.x 23278103

[B19] Liao K. H. Tan Y.-M. Conolly R. B. Clewell H. J. Gargas M. L. Andersen M. E. (2007). Bayesian estimation of pharmacokinetic and pharmacodynamic parameters in a mode-of-action based cancer risk assessment for chloroform. Risk Anal. 27 (6), 1535–1551. 10.1111/j.1539-6924.2007.00987.x 18093051

[B20] Liberacki A. B. (1999). Preliminary results. Midland, Michigan: The Dow Chemical Company. (As cited in Carney and Liberacki 1999).3-chloroally alcohol: Developmental toxicity probe study in CD rats

[B21] Lu X. T. Ma Y. Wang C. Zhang X. F. Jin D. Q. Huang C. J. (2012). Cytotoxicity and DNA damage of five organophosphorus pesticides mediated by oxidative stress in PC12 cells and protection by vitamin E. J. Environ. Sci. Health B 47 (5), 445–454. 10.1080/03601234.2012.663312 22424070

[B22] Nakamura J. Mutlu E. Sharma V. Collins L. Bodnar W. Yu R. (2014). The endogenous exposome. DNA Repair 19, 3–13. 10.1016/j.dnarep.2014.03.031 24767943 PMC4097170

[B23] Nakamura J. Nakamura M. (2020). DNA-protein crosslink formation by endogenous aldehydes and AP sites. DNA Repair 88, 102806. 10.1016/j.dnarep.2020.102806 32070903 PMC7192481

[B24] National Research Council (2007). Toxicity testing in the 21st century: A vision and a strategy. Washington, DC: The National Academies Press. 10.17226/11970

[B25] O'Byrne K. J. Dalgleish A. G. (2001). Chronic immune activation and inflammation as the cause of malignancy. Br. J. Cancer 85 (4), 473–483. 10.1054/bjoc.2001.1943 11506482 PMC2364095

[B26] OECD (2002). Guidance document on the recognition, assessment and use of clinical signs as human endpoints for experimental animals used in safety evaluation, OECD series on testing and assessment, No. 19. Paris: OECD Publishing. 10.1787/9789264078376-en

[B27] OECD (2016). Test No. 474: Mammalian erythrocyte micronucleus test, OECD guidelines for the testing of chemicals, section 4. Paris: OECD Publishing. 10.1787/9789264264762-en

[B28] OECD (2020). Test No. 488: Transgenic rodent somatic and germ cell gene mutation assays, OECD guidelines for the testing of chemicals, section 4. Paris: OECD Publishing. 10.1787/9789264203907-en

[B29] Ohno Y. Ormstad K. Orrenius S. (1985). Mechanism of allyl alcohol toxicity and protective effects of low-molecular-weight thiols studied with isolated rat hepatocytes. Toxicol. Appl. Pharmacol. 78, 169–179. 10.1016/0041-008x(85)90281-9 2930914

[B30] Pastino G. M. Conolly R. B. (2000). Application of a physiologically based pharmacokinetic model to estimate the bioavailability of ethanol in male rats: Distinction between gastric and hepatic pathways of metabolic clearance. Toxicol. Sci. 55, 256–265. 10.1093/toxsci/55.2.256 10828256

[B31] Perleberg U. R. Keys D. A. Fisher J. W. (2004). Development of a physiologically based pharmacokinetic model for decane, a constituent of jet propellent-8. Inhal. Toxicol. 16, 771–783. 10.1080/08958370490490473 16036747

[B32] Reitz R. H. Fox T. R. Quast J. F. (1982). Mechanistic considerations for carcinogenic risk estimation: Chloroform. Environ. Health Perspect. 46, 163–168. 10.1289/ehp.8246163 7151758 PMC1569040

[B33] Simmons S. O. Fan C.-Y. Ramabhadran R. (2009). Cellular stress response pathway system as a sentinel ensemble in toxicological screening. Toxicol. Sci. 111, 202–225. 10.1093/toxsci/kfp140 19567883

[B34] Swenberg J. A. Fryar-Tita E. Jeong Y. C. Boysen G. Starr T. Walker V. E. (2008). Biomarkers in toxicology and risk assessment: Informing critical dose-response relationships. Chem. Res. Toxicol. 21 (1), 253–265. 10.1021/tx700408t 18161944

[B35] Takahashi S. Ikeda Y. Kimoto N. Okochi E. Cui L. Nagao M. (2000). Mutation induction by mechanical irritation caused by uracil-induced urolithiasis in Big Blue rats. Mutat. Res. 447 (2), 275–280. 10.1016/s0027-5107(99)00217-1 10751611

[B36] USEPA (2005). Guidelines for carcinogen risk assessment. Available at: EPA/630/P=03/001F/ .

[B37] USEPA (2001). Toxicological review of chloroform (CAS No. 67-66-3). In Support of Summary Information on the Integrated Risk Information System (IRIS). EPA/635/R-01/001.

[B38] van Wesenbeeck I. J. Knowles S. (1999). Groundwater monitoring for 1,3-dichloropropene in high fumigant use areas of North America and Europe. Pest Manag. Sci. 75, 2278–2282. 10.1002/ps.5398 PMC6767602 30843340

[B39] Wasenbeeck I. J. Knowles S. (2019). Ground water monitoring for 1,3-dichloropropene in high fumigant use areas of North America and Europe. Pest Manag. Sci. 75, 2278–2282. 10.1002/ps.5398 30843340 PMC6767602

[B40] Wolf D. C. Cohen S. M. Boobis A. R. Dellarco V. L. Fenner-Crisp P. A. Moretto A. (2019). Chemical carcinogenicity revisited 1: A unified theory of carcinogenicity based on contemporary knowledge. Regul. Toxicol. Pharmacol. 103, 86–92. 10.1016/j.yrtph.2019.01.021 30634023

[B41] Wolf D. C. Butterworth B. E. (1997). Risk assessment of inhaled chloroform based on its mode of action. Toxicol. Pathol. 25, 49–52. 10.1177/019262339702500110 9061851

[B42] Young M. (2020). *In vivo* mutation assay at the cII locus and *in vivo* micronucleus assay in big blue® transgenic F344 rats with chloroallyl alcohol via drinking water. BTL. BioReliance Study Number AF97GE.171.

[B43] Yuan J. (1993). Modeling blood/plasma concentrations in dosed feed and dosed drinking water toxicology studies. Toxicol. Appl. Pharmacol. 119, 131–141. 10.1006/taap.1993.1052 8470117

