# Supplementary material for: PBPK modeling to evaluate maximum tolerated doses: A case study with 3-chloroallyl alcohol
Source: Front Pharmacol. 2023 Feb 22;14:1088011. doi: 10.3389/fphar.2023.1088011 (PMC9992188; doi:10.3389/fphar.2023.1088011)
Supplement: Supplementary file 1 [file Table1.DOCX]

**Appendix A**
